# Changes in Medical Schools

**Published:** 1859-09

**Authors:** 


CHANGES IN MEDICAL SCHOOLS.—In the Medical College of Ohio, Dr. J. Wood has resigned his chair, and two new professorships have been instituted in its stead. That of Clinical Medicine has been filled by the appointment of Professor Graham, and Dr. B. F. Richardson fills that of Diseases of Women and Children.

The vacancies in the New York Medical College have been filled by the transfer of Dr. Peaslee to the Chair of Obstetrics, and by the appointment of Dr. Austin Flint, Jr., to the Professorship of Physiology and Microscopical Anatomy. Dr. James Bryan occupies the Chair of Anatomy, and Dr. Meier that of Surgical Anatomy and General Pathology.

In the Shelby Medical College, Dr. Henry Erni succeeds Dr. Currey in the Chair of Chemistry.

Dr. John E. Crowe has been appointed Demonstrator of Anatomy in the Medical Department of the University of Louisville; and Dr. Demmé fills the same post in the Pennsylvania Medical College.

The Faculty of Medicine of Paris recently presented the following candidates for the Chairs of Physiology and Pharmacy. For the former, Drs. Longet and Béclard, and for the latter, MM. Régnault, Leconte, and Orfila.

The successful candidates were MM. Longet and Régnault.

